# The inhibitory potential of three scorpion venom peptides against multidrug-resistant *Klebsiella pnemoniae*

**DOI:** 10.3389/fmicb.2025.1569719

**Published:** 2025-05-30

**Authors:** Rosa Giugliano, Roberta Della Marca, Annalisa Chianese, Alessandra Monti, Federica Donadio, Emanuela Esposito, Nunzianna Doti, Carla Zannella, Massimiliano Galdiero, Anna De Filippis

**Affiliations:** ^1^Department of Experimental Medicine, University of Campania "Luigi Vanvitelli", Naples, Italy; ^2^Institute of Biostructures and Bioimaging (IBB), National Research Council (CNR), Naples, Italy; ^3^Institute of Applied Sciences and Intelligent Systems (ISASI), Naples Cryo Electron Microscopy Laboratory - EYE LAB, National Research Council (CNR), Naples, Italy; ^4^Complex Operative Unit of Virology and Microbiology, University Hospital of Campania "Luigi Vanvitelli", Naples, Italy

**Keywords:** scorpion venom peptide, antibacterial activity, multi-drug resistant bacteria, *Klebsiella pneumoniae*, synergistic action

## Abstract

**Background:**

The rise of drug-resistant bacterial infections, particularly those caused by *Klebsiella pneumoniae* underscores the urgent need for novel therapeutic candidates. Hospital-acquired infections from *K. pneumoniae* carbapenemase (KPC)-producing bacteria pose a serious health threat, particularly impacting the respiratory and urinary tracts. This study investigates the antibacterial efficacy of three antimicrobial peptides, called pantinins, derived from *Pandinus imperator* scorpions’ venom against *K. pneumoniae* and various KPC-producing clinical isolates.

**Methods:**

Non-toxic concentrations were established for each peptide using MTT method. Antibacterial activity was performed through the liquid microdilution assay to assess the minimum inhibitory concentration (MIC) and the minimal bactericidal concentration (MBC). A time-kill test was conducted by recording the colonies forming units (CFUs) at several times and compared with those of the untreated bacteria. Finally, the effect of peptides on the gene expression of virulence factors of *K. pneumoniae* was evaluated through qPCR.

**Results:**

Antibacterial assays demonstrated bactericidal effects for pantinin-1 and pantinin-2 within 1 h, while pantinin-3 displayed bacteriostatic properties. Noteworthy, MIC values ranged from 6 to 25 μM for *K. pneumoniae* and from 25 to 50 μM for KPC strains. Pantinins exert their antimicrobial effect through a membranolytic mechanism, directly interacting with outer membrane lipopolysaccharides (LPS), as evidenced by circular dichroism (CD) spectra and scanning electron microscopy (SEM). In addition, qPCR showed a reduction in virulence genes expression, suggesting the antibacterial effect of peptides also at transcriptional level.

**Conclusion:**

These findings support pantinins as promising candidates for treating multidrug-resistant *K. pneumoniae* infections.

## 1 Introduction

Antibiotic resistance is a constantly increasing phenomenon and threatens global public health. A recent study estimated that over a million deaths were caused by bacterial antimicrobial resistance (AMR) predicting that more than 39 million people could die over the next 25 years (GBD 2021 Antimicrobial Resistance Collaborators, 2024). The indiscriminate and prolonged use of antibiotics, especially in developing countries, has contributed to the progress and spread of pharmaco-resistant microorganisms in different fields, such as agriculture, medicine, and veterinary (Huan et al., 2020). ESKAPE pathogens as *Enterococcus faecium*, *Staphylococcus aureus*, *Klebsiella pneumoniae*, *Acinetobacter baumannii*, *Pseudomonas aeruginosa,* and *Enterobacter* spp., are multi-antibiotic-resistant bacteria (Miller and Arias, 2024). This makes the treatment of infections mediated by them complicated, contributing to increased disease development and mortality. For this reason, ESKAPE pathogens have been designated by WHO as “priority status “(De Oliveira et al., 2020). Multi-drug resistant (MDR) bacteria certainly include extended-spectrum beta-lactamase-producing *K. pneumoniae* (ESBL), carbapenem-resistant *Escherichia coli* (CRE), *Enterobacteriaceae*, and multidrug-resistant *A. baumannii* (MRAB) (Medina and Pieper, 2016). Indeed, in the European Union and China, most clinical infections (64–87.8%) are caused by CRE and carbapenem-resistant *K. pneumoniae* (CRKP) (Zhang et al., 2022). *K. pneumoniae* is a nonmobile Gram-negative opportunist pathogen belonging to the *Enterobacteriaceae* family, and it is mainly responsible for nosocomial infections of the respiratory and urinary tracts, representing the second most common cause of septicemia among bacteria belonging to this family (Zhu et al., 2021; Karampatakis et al., 2023). In the present emergency, a new strategy is needed to counter the spread of antibiotic resistance. Antimicrobial peptides (AMPs), naturally occurring molecules, play a key role in the innate immune response in a wide range of organisms, from bacteria to humans. AMPs, short peptides typically of 10–50 amino acids, show broad-spectrum antimicrobial activity against bacteria, fungi, viruses, and even tumor cells (Gagandeep et al., 2024). AMPs are known for their ability to rapidly target and disrupt microbial cell membranes, leading to cell death. This mechanism makes them highly effective, especially against antibiotic-resistant pathogens where traditional therapies may fail (Park et al., 2018; Huan et al., 2020; Luong et al., 2020; Memariani et al., 2020; Agarwal et al., 2016). To date, more than 300 AMPs have been discovered. Notably, the US Food and Drug Administration (FDA) has already approved 7 of them, while ca. 36 are in the study phase, 7 in the preclinical phase, and 29 in the clinical investigation phase (Panayi et al., 2024). Scorpion venom has emerged as a remarkable source of AMPs with unique structures and potent bioactivities (Harrison et al., 2014). Scorpion peptides include three different groups: (i) cysteine-containing peptides with disulfide bridges (DBPs); (ii) cysteine-free peptides non-disulfide-bridges (NDBPs); (iii) peptides rich in proline and glycine amino acids. In this study, three NDBPs (pantinin 1, pantinin 2, and pantinin 3), secreted by the scorpion *Pandinus imperator* (Harrison et al., 2014) were analyzed for their antimicrobial efficacy against the reference strain of *K. pneumoniae* and multiple clinical isolate strains. The investigation included an assessment of the kinetics of action and structural conformational changes of peptides interacting with bacterial membranes. Additionally, the ability of peptides to eradicate *K. pneumoniae* biofilms was examined. Lastly, the study explored the potential of combination therapy using these peptides alongside antibiotics to lower the required antibiotic concentrations as a strategy to mitigate drug resistance.

## 2 Methods

### 2.1 Peptide synthesis

Peptides were synthesized amidated at the C-terminal end using the solid-state Fluorenylmethyloxycarbonyl (Fmoc) strategy on the SYRO I automatic multiple peptide synthesizer (Biotage), following established protocols (Caporale et al., 2017; Caporale et al., 2018). The synthesized peptides were purified using reversed-phase high-performance liquid chromatograph (RP-HPLC) on a WATERS 2545 preparative system (Waters, Milan) with a UV/Vis detector (WATERS 2489). The identity and purity of peptides were assessed by liquid chromatography-mass spectrometry (LC–MS) analysis using an LTQ XL™ Linear Ion Trap Mass Spectrometer (Thermo Scientific™), with a Waters xBridge C18 column (5 μm, 2.1 × 50 mm), applying a linear gradient of CH3CN/0.05% TFA in 0.05% TFA/H2O from 10 to 80% in 10 min, at a flow rate of 0.2 mL/min. All peptides achieved a purity greater than 95%.

### 2.2 Stability of peptides in serum

Peptide stability in serum was performed as previously reported (Russo et al., 2021). Briefly, peptides were diluted in serum to a final concentration of 2.0 mg/mL and incubated at 37°C. Aliquots of each peptide taken at different times up to 16 h were analyzed by RP-HPLC on an Aeris PEPTIDE XB-C18 column (2.1 × 100 mm, 3.6 μm). Peptide concentrations in solution were determined from RP-HPLC peak areas compared to peak areas obtained at t0 (set as 100%).

### 2.3 Circular dichroism (CD) measurements

CD spectra of peptides at 50 μM were recorded from 260 to 190 nm, using the JASCO-705 CD spectrophotometer (JASCO) at room temperature. Titrations experiments were performed by fixing the concentration of each peptide at 50 μM and adding increasing concentrations of TFE or LPS. Each spectrum was corrected by subtracting the appropriate buffer spectrum without the peptide. Sample solutions were prepared using 5.0 mM sodium phosphate buffer at pH 7.4. All spectra were recorded using a 0.1 cm path length cuvette with a scan speed of 50 nm/min, response time of 1 s, and bandwidth of 1 nm. Each spectrum was collected by averaging 3 spectra. The CD intensity (mdeg) was expressed as the mean residue ellipticity ([ɵ] *10^−3^ (deg*cm^2^*dmol^−1^)) calculated with reference to the total amino acid content.

### 2.4 Cytotoxicity on HaCaT cell

Peptides cytotoxicity was evaluated on the HaCaT cell line (human keratinocytes cells) obtained from the CLS–Cell Lines Service (Eppelheim, Germany). The cell monolayer was treated with different peptide concentrations from 3 to 100 μM for 24 h. Then, methylthiazolyldiphenyl-tetrazolium bromide (MTT) (Sigma-Aldrich, St. Louis, MO, USA) assay was performed as already reported (Ambrosino et al., 2023). Untreated cells represented positive control (CTRL+), while cells treated with 100% DMSO represented negative control (CTRL−). Absorbance was recorded at 570 nm, and cell viability was calculated with Equation 1:


(1) $$\%\mathrm{of}\;\mathrm{cell}\;\mathrm{viability}=\frac{\mathrm{absorbance}\;\mathrm{of}\;\mathrm{treated}\;\mathrm{cells}}{\mathrm{absorbance}\;\mathrm{of}\;\mathrm{untreated}\;\mathrm{cells}}\;x\;100$$

### 2.5 Antibacterial assay by the broth microdilution method

Antibacterial activity of peptides was conducted against a panel of bacterial strains both Gram-positive (*Staphylococcus aureus* ATCC 6538, *Enterococcus faecalis* ATCC 29212, *Listeria monocytogenes* MB 677), and Gram-negative (*E. coli* ATCC 11229, *K. pneumoniae* ATCC 10031, *Pseudomonas aeruginosa* O1, *Burkordelia cepacea* LGM16656). The analysis has been extended to clinical isolates of *K. pneumoniae* carbapenemases producers isolated and characterized at the Microbiology Laboratory of the University Hospital “Luigi Vanvitelli,” Naples, Italy, using a BD Phoenix M50 instrument (Becton, Dickinson and Company, Franklin Lakes, NJ, USA) and listed in Table 1. The test was carried out as reported elsewhere (Folliero et al., 2022). The final bacterial concentration inoculated in the 96-well plate was 1 × 10^5^ CFU/mL. Vancomycin (5 μg/mL) and meropenem (10 μg/mL) (Burlington, MA, USA) were used as positive control (CTRL+) against Gram-positive and Gram-negative bacteria, respectively.

**Table 1:** Characteristics and source of *K. pneuomiae* isolates.

Bacterial strain	Carbapenemase	Source
*K. pneumoniae* 1711	KPC (class A)	Rectal swab
*K. pneumoniae* 1745	KPC (class A)	Ulcer
*K. pneumoniae* 1746	VIM (class B)	Rectal swab
*K. pneumoniae* 311	OXA-48 (class D)	Rectal swab

The type of carbapenemase produced refers to the classification of Ambler.

#### 2.5.1 MIC and minimum bactericidal concentration (MBC)

The concentration of peptides inhibiting 95% bacterial growth was recorded as MIC. After setting the MIC value, 50 μL were taken from the 2MIC, 1MIC, and ½ MIC and spotted on MHA. The plates were incubated for 18 h at 37°C. The number of colony-forming units was calculated, and MBC was recorded as the lowest concentration of peptide capable of killing 99.9% of the bacterial load after 18 h of incubation.

#### 2.5.2 Time killing assay

The same conditions as the MIC were used for the time to kill assay (Buonocore et al., 2023). The effect of peptides was observed at defined time intervals (0, 1, 2, 3, 6 and 18 h). At each set time, 10 μL were taken from the wells, i.e., 4x MIC, 2x MIC, 1x MIC e ½ x MIC, and 10-fold serial dilutions in PBS were performed. 10 μL of each dilution was spotted on MHA and allowed to dry under the biological hood. Then, the plates were incubated at 37°C for 18 h, and the CFUs/mL were calculated by counting the number of colonies in the treated samples and those present in the untreated bacterium (CTRL−).

### 2.6 Scanning electron microscopy (SEM)

Scanning electron microscopy (SEM) was used to observe morphological changes. *K. pneumoniae* was treated as described above in the MIC assay. After 1 h, the treatment was stopped, and the bacterial cells were recovered and centrifuged at 500 rpm for 15 min. The pellet was washed 2 times with PBS and fixed with 4% formaldehyde O/N at 4°C. The cells were dehydrated with increasing concentrations of ethanol (25, 50, 75, 95, 100%). For each sample, 2 μL were dropped onto a cleaned silicon wafer and air-dried at room temperature. The images ware obtained by using dual beam FIB-SEM Aquilos 2 by ThermoFisher Scientific. The acquisition parameters were Current 25 pA, Voltage 3 kV, Working Distance 5.2 mm, Field of View 41.4 μm, Stage Tilt 0° and Magnification 5,000X.

### 2.7 Checkerboard tests

To assess the combined effect of peptides and antibiotics, a checkerboard test was performed. An inoculum of *K. pneumoniae* at 2 × 10^5^ CFU/mL was used for the assay. Serial dilutions of peptides and meropenem were executed, and 10 μL of peptide and antibiotic were added into the 96-well plate, individually and/or in combination. The plate was incubated for 24 h to 37°C and the combinatorial effect was calculated as an index of fractional inhibitory concentration (FICI) with the following formula (Giugliano et al., 2023).


$$\begin{array}{l} \mathrm{FICI}=\\ \frac{\mathrm{MIC}\;\mathrm{meropenem in combination}}{\mathrm{MIC}\;\mathrm{meropenem}}+\frac{\mathrm{MIC}\;\mathrm{peptide in combination}}{\mathrm{MIC}\;\mathrm{peptide}} \end{array}$$

- FICI ≤ 0.5, synergy.
- 0.5 < FICI ≤ 1.0, partial synergy.
- < FICI ≤ 4.0, no interaction.
- FICI > 4.0, antagonism.

### 2.8 Biofilm initial attachment assay

An inoculum of *K. pneumoniae* O/N was diluted to 0.1 OD_600_. 100 μL of bacterial suspension and 100 μL of peptides at different concentrations were plated in the 96 well. After 2 h of incubation at 37°C, the plate was emptied and washed 2 times with PBS. Then, the plate was filled with 95 μL of fresh medium and 5 μL of MTT and incubated at 37°C for 3 h. The measurement was made by reading the plate at 570 nm and calculated according to the following formula:


$$\begin{array}{l} \%\mathrm{Attachment inhibition}=100\times [1-{OD}_{570}\;\mathrm{of the treated}\\ \mathrm{bacteria}/{OD}_{570}\;\mathrm{of the untreated bacteria}] \end{array}$$

### 2.9 Biofilm inhibition

A single colony of *K. pneumoniae* was inoculated in 5 mL of MH supplemented with 2% glucose (MHG) and incubated for 20 h at 37°C. The bacterial suspension was diluted up to 0.1 OD600, and 100 μL were added to the 96 well. Peptides were added at different concentrations for 20 h. After treatment, the plate was emptied and washed 2 times with PBS to remove the suspended cells. Inhibition of biofilm formation was calculated using the violet crystal method (CV). 100 μL of CV at 0.05 were added to each well, and the plate was left at room temperature (RT) for 40 min (min). Next, the wells were washed with sterile water to remove the excess dye, and 100 μL of ethanol (EtOH) were added for another 40 min. Lastly, the plate was measured at 570 nm at the Tecan microplate reader. The percentage of inhibition was calculated with the following formula:


$$\begin{array}{l} \%\mathrm{Biofilm inhibition}=100\times \;[1-{OD}_{570}\;\mathrm{of the treated}\\ \mathrm{bacteria}/{OD}_{570}\;\mathrm{of the untreated bacteria}] \end{array}$$

### 2.10 Biofilm degradation

A bacterial inoculum of *K. pneumoniae* was adjusted to 10^8^ CFU/mL (0.2 OD_600_)_,_ and 200 μL of this was dispensed in the 96-well plate (Giugliano et al., 2023). The plate was incubated for 48 h at 37°C to allow the formation of the mature biofilm. Later, the supernatant was aspirated, and the biofilm was washed 2 times with PBS. The plate was filled with 200 μL of peptides and incubated for 24 h at 37°C. Finally, the plate was emptied, washed twice with PBS, and processed using the CV method as described above. The percentage of biofilm degradation was calculated according to the following formula:


$$\begin{array}{l} \%\mathrm{Biofilm degradation}=100\times [1-{OD}_{570}\;\mathrm{of the treated}\\ \mathrm{bacteria}/{OD}_{570}\;\mathrm{of the untreated bacteria}] \end{array}$$

### 2.11 Gene expression of virulence factors

To assess the expression of bacterial genes, the MIC test was performed as described above. The treatment was blocked after 1 h, and total RNA was extracted with TRIzol® reagent (Thermo Fisher, Waltham, MA, USA), and quantified at Nanodrop (nanodrop 2000, Thermo Fisher Scientific, Waltham, MA, USA). 1 μg of RNA was retrotranscribed to cDNA using the 5x all-in-One RT mastermix (Applied Biological Materials, Richmond, Canada). Real-Time PCR was performed in duplicate using the Insta Q96 (− 6.0) thermocycler. Briefly, 2 μL of cDNA were amplified in a 20 μL of reaction using BlasTaq 2 qPCR mastermix (Applied Biological Materials, Richmond, Canada) and 0.1 μM of primers. The mRNA levels of cells treated with peptides were compared with untreated cells (control) and normalized with respect to the internal housekeeping gene 16S. The 2-ΔΔCT method was applied to calculate the threshold cycle (Ct) difference between the target and housekeeping genes. Primers were purchased by Eurofins (Vimodrone, Milan, Italy) and their sequences are reported in Table 2.

**Table 2:** Sequences of the primers used for the real-time PCR.

Genes	Forward sequence	Reverse sequence
*galF*	GCTATAACCTCGCCGCTATG	GCTCGTCCGGTTTTTCAATA
*ompW*	TTTCCGATCTGAGCCTGAAG	AGACGGGTGCTGACTTTCTG
*fiu*	GGACCGTGCATTTTAAAGGA	GGCTACCGTAGTGTCGCAAT
*16S*	ATTTGAAGAGGTTGCAAACGAT	TTCACTCTGAAGTTTTCTTGTGTTC

### 2.12 Statistical analysis

All tests were performed in triplicate and expressed as mean ± Standard Deviation (SD), One-way ANOVA and Two-way ANOVA, both followed by Dunnett’s test for multiple comparisons, and graphs were generated using GraphPad Prism ver. 9.5.1 for macOS (GraphPad Software, San Diego, CA, USA)^1^.

## 3 Results

### 3.1 Pantinin peptides production and characterization

Pantinin peptides were synthesized by the Fmoc method (Merrifield, 1963) and subsequently purified, achieving more than 95% purity, as confirmed by LC–MS analysis. Each peptide showed a distinct single peak with a retention time of around 12 min (Supplementary Figure S1–S3), and MS results were consistent with the theoretical values displaying m/z signals of 1547.08 ([M + H]^+^) and 773.90 ([M + 2H]^2+^) for Pantinin 1; 1403.98 ([M + H]^+^), 1426.00 ([M + Na]^+^) and 702.81 ([M + 2H]^2+^) for Pantinin 2 and 1492.04 ([M + H]^+^), 1513.04 ([M + Na]^+^) and 746.36 ([M + 2H]^2+^) for Pantinin 3 (Table 3).

**Table 3:** Retention times (tR), amino acid sequences, and theoretical and experimental monoisotopic masses (MWs) of synthetic pantinin peptides.

Entry	Sequence	t_R_ (min)	MW (theoretical)	MW (experimental)
Pantinin 1	GILGKLWEGFKSIV-NH2	12.08	1544.90	1545.11
Pantinin 2	IFGAIWKGISSLL-NH2	12.26	1402.82	1402.98
Pantinin 3	FLSTIWNGIKSLL-NH2	12.29	1489.86	1490.14

The experimental masses agree well with the theoretical ones.

### 3.2 CD analysis

**Figure 1:**
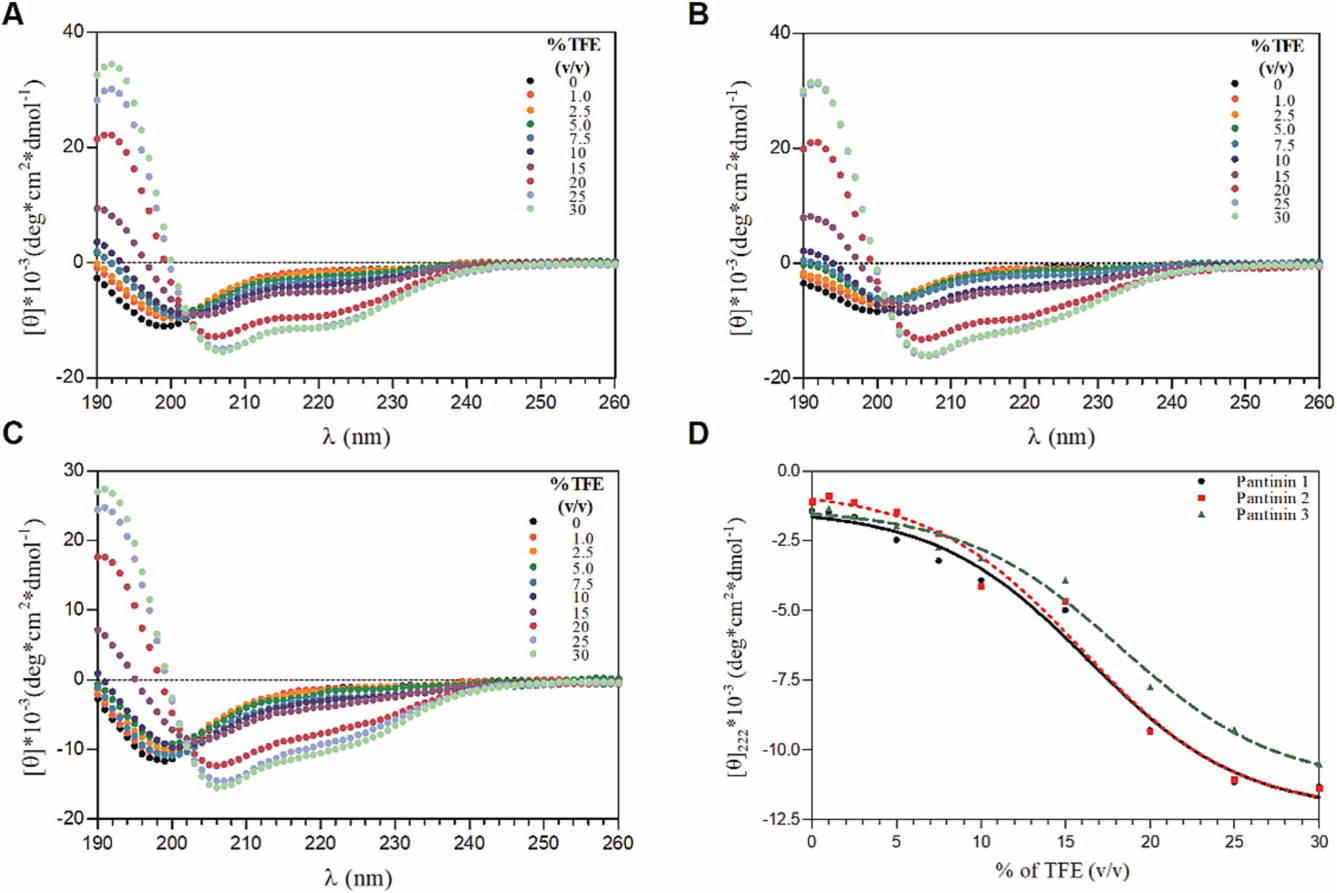
CD-titration experiments of pantinin peptides with TFE **(A-C)**. Each peptide at 50 μM was treated with increasing percentage of TFE (0 ÷ 30%, v/v). **(D)** The mean residue ellipticity at 222 nm as a function of the % of TFE. Sample solutions were prepared using 5.0 mM sodium phosphate buffer at pH 7.4. CD intensity (mdeg) was expressed as mean residue ellipticity ([ɵ]*10^−3^ (deg*cm^2^*dmol^−1^)) calculated referring to the total amino acid content.

The conformational behavior of synthetic pantinin peptides in aqueous solution and in TFE/H_2_O mixtures was evaluated by Far-UV CD spectroscopy. As shown in Figures 1A–C (see black lines), the peptides adopt a predominantly disordered state in the aqueous buffer, showing a minimum around 200 nm and a negative value at 190 nm. However, in solutions containing 30% TFE (v/v) (Figures 1A–C, see light green lines), the presence of large minima of residual molar ellipticity at 208 and 220 nm and a maximum at 192 nm in the CD spectra, shows a clear helical content in all three tested peptides, consistent with previous results (Crusca et al., 2018). Here, the helix propensity for the three synthetic peptides was comparatively assessed by CD titration experiments with TFE (Figures 1A–C). The plot of residual ellipticities at 220 nm as a function of percentage TFE shows that the TFE titration curves of all three peptides are sigmoidal, each with a single transition point (Figure 1D), consistent with a folding process two-state, from the unfolded state to the alpha-helical state. This two-state folding is also supported by the presence of a single isodichroic point at 202 nm in the CD spectra for all TFE concentrations (Figures 1A–C). Furthermore, it is observed that unlike pantinin 3, pantinin 1 and pantinin 2 reach saturation already at 25% TFE, indicating a greater propensity of the two peptides to adopt helical conformations compared to pantinin 3, in line with data reported in literature (Crusca et al., 2018).

**Figure 2:**
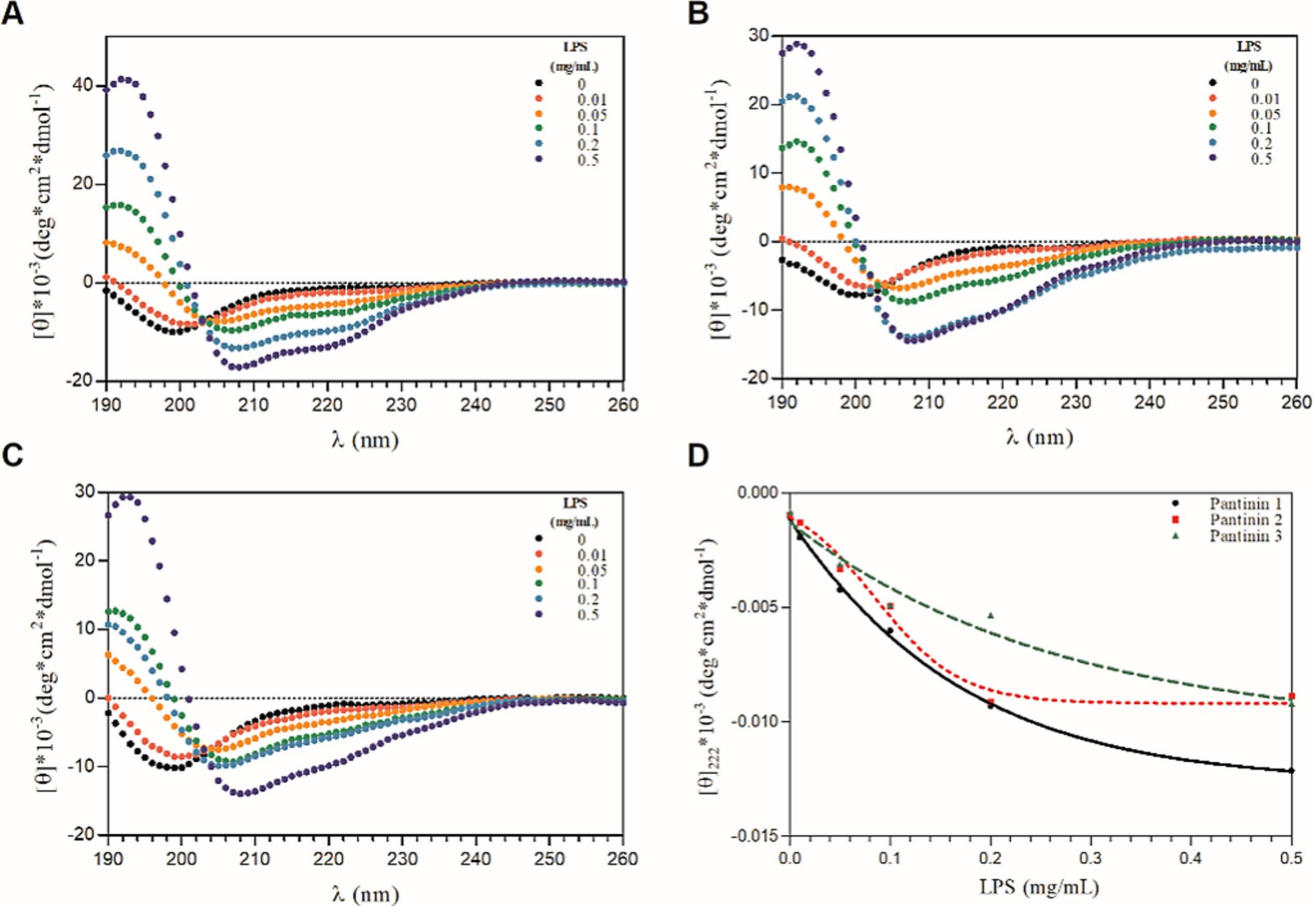
CD-titration experiments of pantinin peptide with LPS **(A-C)**. Each peptide at 50 μM was treated with increasing concentrations of LPS (0 ÷ 0.5 mg/mL). **(D)** The mean residue ellipticity at 222 nm as a function of the concentrations of LPS (0 ÷ 0.5 mg/mL). Sample solutions were prepared using 5.0 mM sodium phosphate buffer at pH 7.4. CD intensity (mdeg) was expressed as mean residue ellipticity ([ɵ]*10^−3^ (deg*cm^2^*dmol^−1^)) calculated referring to the total amino acid content.

The conformation of pantinin peptides was also evaluated in the presence of LPS, the main component of the outer membrane of most Gram-negative bacteria (Di Lorenzo et al., 2022), by performing CD titration experiments with different concentrations of LPS. As shown in Figure 2A–C, the addition of LPS to peptide solutions produces a significant change in the secondary structure of the peptides, already at the lowest concentrations of LPS tested (Figure 2D), indicating a direct and strong interaction of the peptides with lipopolysaccharides. The presence of CD bands around 208 and 222 nm indicates that the peptides fold into *α*-helix upon interaction with LPS. As observed in TFE, even in the presence of LPS a local two-state transition is observed (from the unfolded state to the helical structured state), as indicated by the presence of a single isodichroic point at 202 nm in the CD spectra (Figures 2A–C).

### 3.3 Serum stability of pantinin peptides

**Figure 3:**
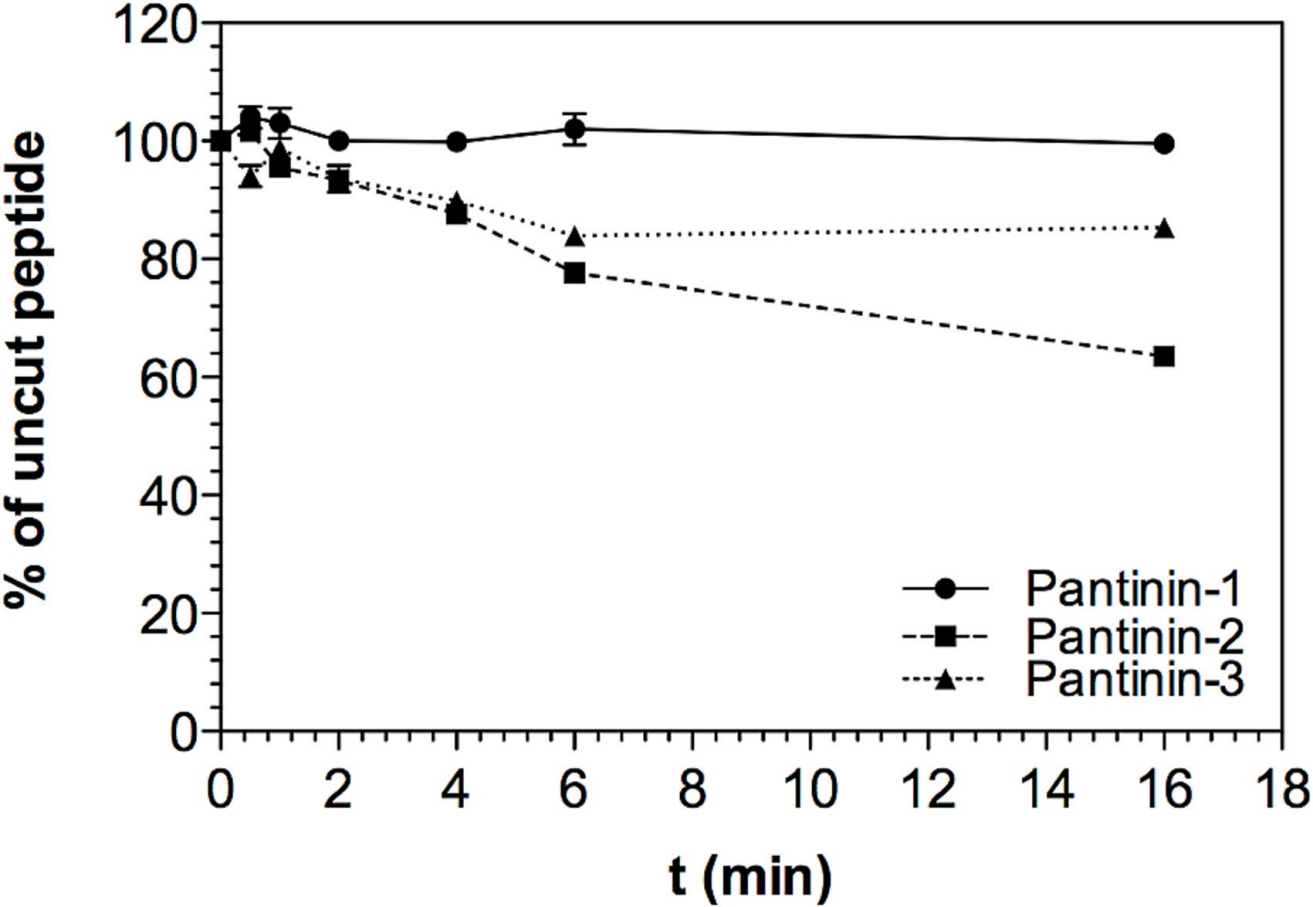
Stability of peptides in serum. Percentage of peptide remaining in solution up to 16 h of incubation in serum, data shown as mean ± SD from experiments performed in duplicates. Peptide concentrations in solution were determined from RP-HPLC peak areas compared to peak areas obtained at t0 (set as 100%).

The stability of pantinin peptides was assessed by exposing them up to 16 h to a serum solution (see Materials and Methods for details). The full-length peptide amounts were calculated relative to the quantities determined at time point zero (see Materials and Methods for details). As shown in Figure 3, the percentage of intact peptide in the presence of the serum slightly decreases over time up to 4 h for the three synthetic peptides. After 4 h of incubation with the serum, pantinin 2 and 3 begin to degrade. However, after 16 h a considerable quantity of each peptide remains in solution, approximately 70 and 80%, respectively (Figure 3). Differently, pantinin 1 is stable in serum in the time interval analysed (Figure 3). The data obtained therefore indicate that the peptides are very stable in serum.

### 3.4 Cytotoxic profile of pantinins

**Figure 4:**
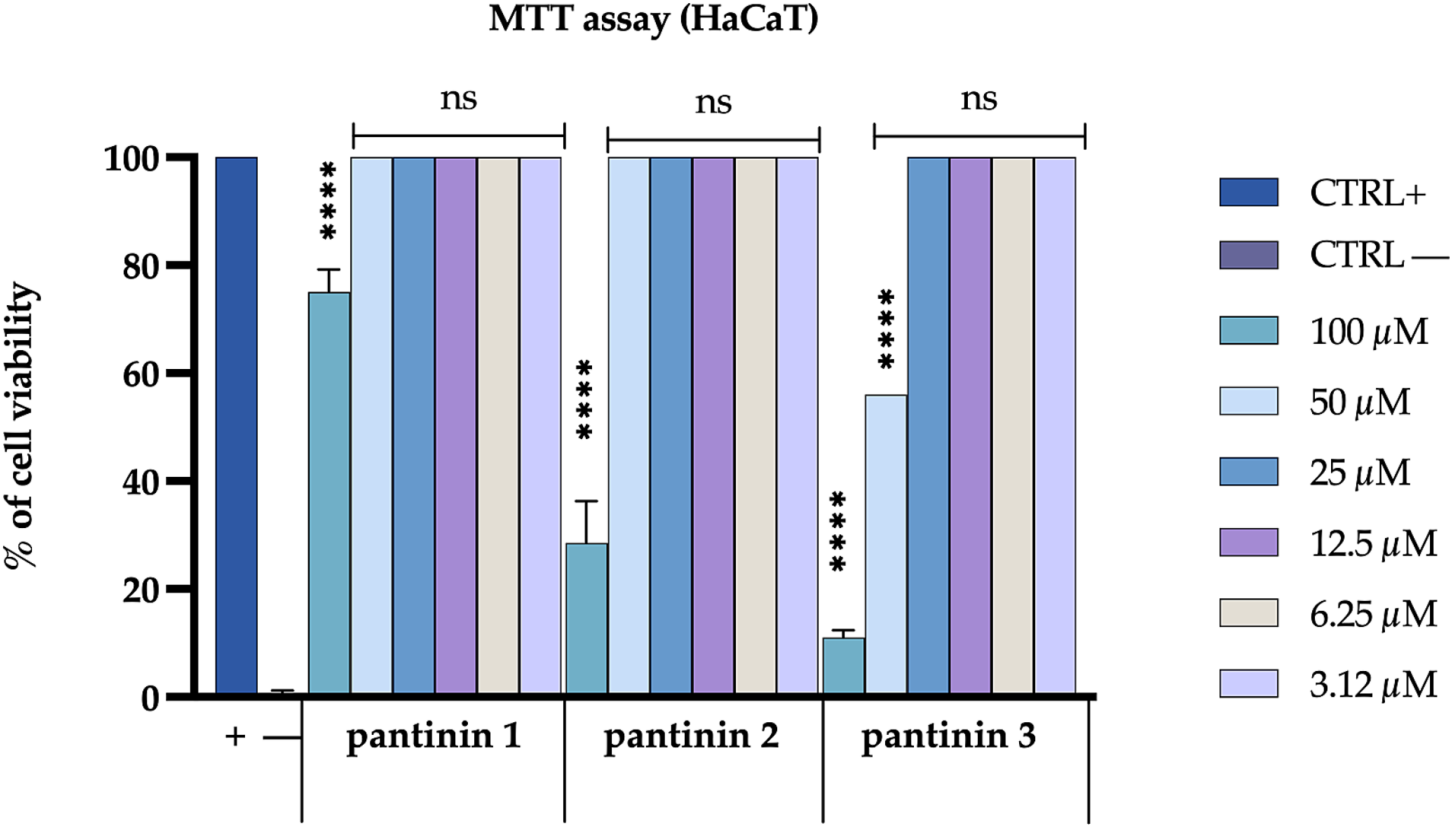
Evaluation of the cytotoxicity of pantinins on the Hacat cell line. Positive control (CTRL+) is represented by untreated cells, while negative control (CTRL−) is represented by cells treated with DMSO (100%). The statistical analysis was done by two-way ANOVA with Dunnett’s test for multiple comparisons. Significances are referred to the untreated cells. **** *p* < 0.0001, ns (not significant).

The cytotoxic effect of the three peptides has been evaluated on the HaCaT cell line. The cell monolayer was treated with different peptide concentrations, ranged from 100 to 3.12 μM. After 24 h of treatment, pantinin 1 exhibited the lowest cytotoxic profile, showing cell viability of approximately 70% at the highest concentration evaluated (100 μM). In comparison, pantinin 2 and pantinin 3 showed 25 and 10% cell viability, respectively, at 100 μM. No effect on viability was observed at other concentrations (Figure 4).

### 3.5 Antibacterial activity of pantinins

Pantinins antibacterial activity against a large panel of bacterial strains was evaluated by MIC assays (Buonocore et al., 2020). All three peptides efficiently inhibited the growth of tested bacteria at concentrations ranging from 6.25 to 50 μM. Among the Gram-positive the best result was against *S. aureus* for pantinin 1 and pantinin 3 with a MIC values of 6.25 μM and 12.5 μM respectively, and against *L. monocytogenes* for pantinin 2 with MIC of 6.25 μM (Table 4). Noteworthy, MICs were very low against *K. pneumoniae*: in detail, we observed inhibition values at 6.25, 12.5, and 25 μM, for pantinin 1, pantinin 2, and pantinin 3, respectively (Table 5).

**Table 4:** Bacterial strains investigated for antibacterial activity.

Minimal inhibitory and bactericidal concentrations (μM)						
Bacterial strain	P1		P2		P3	
	MIC	MBC	MIC	MBC	MIC	MBC
*E. faecalis*	12.5	25	12.5	12.5	25	25
*L. monocytogenes*	6.25	12.5	6.25	12.5	25	-
*S. aureus*	6.25	6.25	12.5	12.5	12.5	-
*P. aeruginosa*	50	-	50	-	50	-
*B. cepacia*	25	25	50	-	50	-
*S. thyphimurium*	50	50	50	50	50	-
*K. pneumoniae*	6.25	12.5	12.5	12.5	25	-
*E. coli*	12.5	25	25	25	50	-

**Table 5:** Antibacterial activity of pantinins against clinical *K. pneumoniae* strains.

Minimal inhibitory and bactericidal concentrations (μM)						
Bacterial strain	P1		P2		P3	
	MIC	MBC	MIC	MBC	MIC	MBC
*K. pneumoniae ATCC 10031*	6.25	12.5	12.5	12.5	25	-
*K. pneumoniae 311*	25	25	-	-	-	-
*K. pneumoniae 1746*	25	-	25	50	50	-
*K. pneumoniae 1745*	25	25	50	50	-	-
*K. pneumoniae 1711*	25	25	50	-	-	-

Therefore, as WHO identified carbapenem-resistant and third-generation cephalosporin-resistant *Klebsiella pneumoniae* as critical priority (World Health Organization, 2024), we conducted additional inhibition tests against several strains of *K. pneumoniae* KPC-producers (Table 5). Pantinin 1 showed the best results with a MIC value of 25 μM against all the tested strains. Values between 25 and 50 μM were recorded for pantinin 2, while the MIC for pantinin 3 was only detected against the strain 1746 (50 μM). Subsequently, to test whether the peptides inhibited growth or killed bacterial cells, the MBC was also investigated against all the clinical isolates. The results showed that pantinin 1 was bactericidal at MIC concentration against all *K. pneumoniae* isolates except for *K. pneumoniae* 1746, where only a bacteriostatic action was highlighted. On the other hand, pantinin 2 had a bactericidal effect against *K. pneumoniae* 1745 and 1746, while pantinin 3 was bacteriostatic against all the strains analyzed (Table 5).

**Figure 5:**
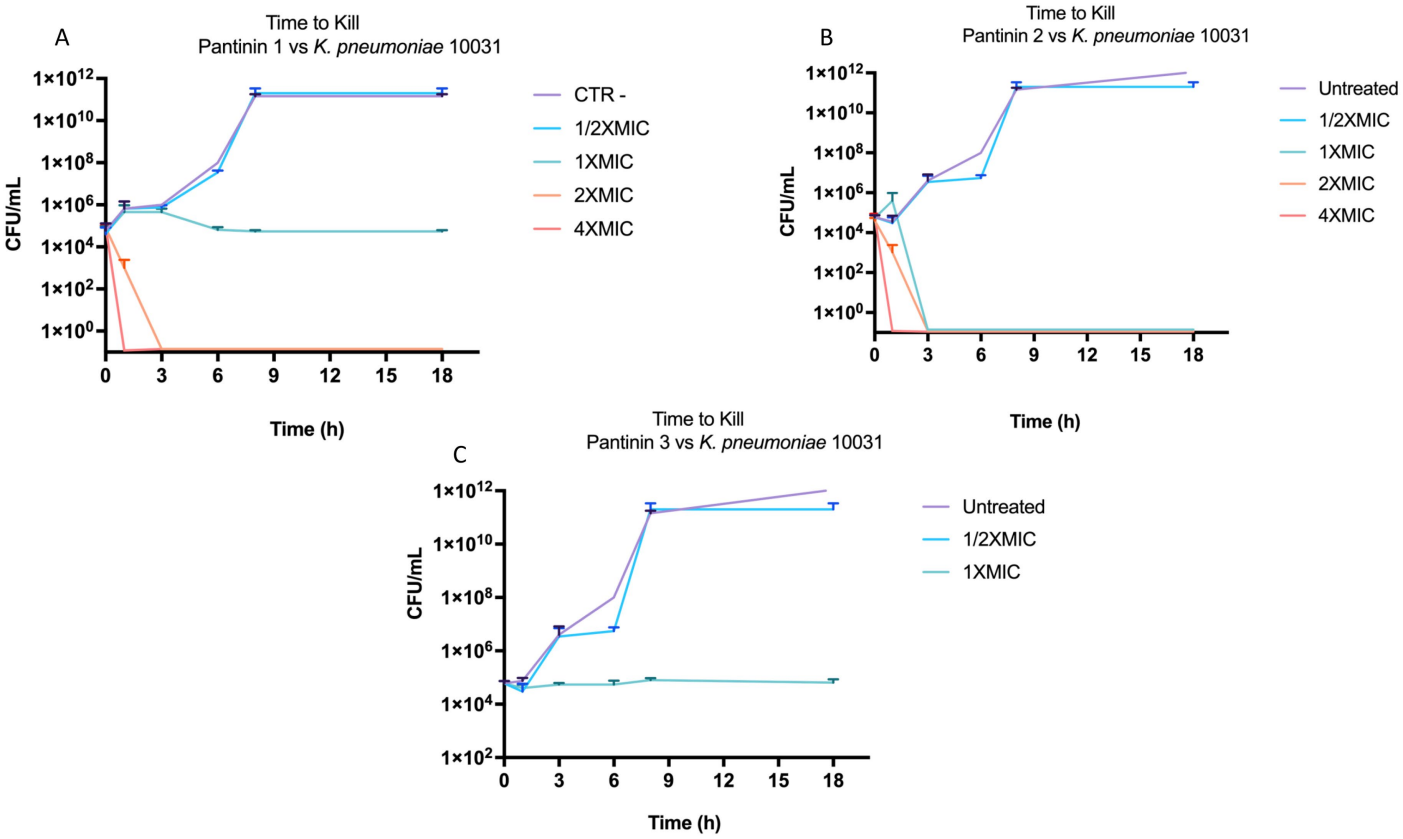
Bactericidal and bacteriostatic effect of pantinins over time against *k. pneumoniae* 10031. **(A)** Time course of pantinin 1, **(B)** pantinin 2, and **(C)** pantinin 3.

The antibacterial action was further deepened by monitoring the kill-time action of pantinins against *K. pneumonia* (Figure 5). The results showed that pantinin 1 eradicated bacterial cells within 1 h at concentrations of 4 × MIC (25 μM) and 2 × MIC (12.5 μM). At the same time, at the MIC value (6.25 μM), it inhibited bacterial replication, assuming a bacteriostatic trend. Similarly, pantinin 2 was bactericidal after 1 h at both 4 × MIC (50 μM) and 2 × MIC (25 μM), and after 2 h at 2 × MIC (12.5 μM). Instead, pantinin 3 had a bacteriostatic effect at the concentration of MIC (50 μM).

### 3.6 Observation of *Klebsiella pneumoniae* bacterial cells under scanning electron microscope

**Figure 6:**
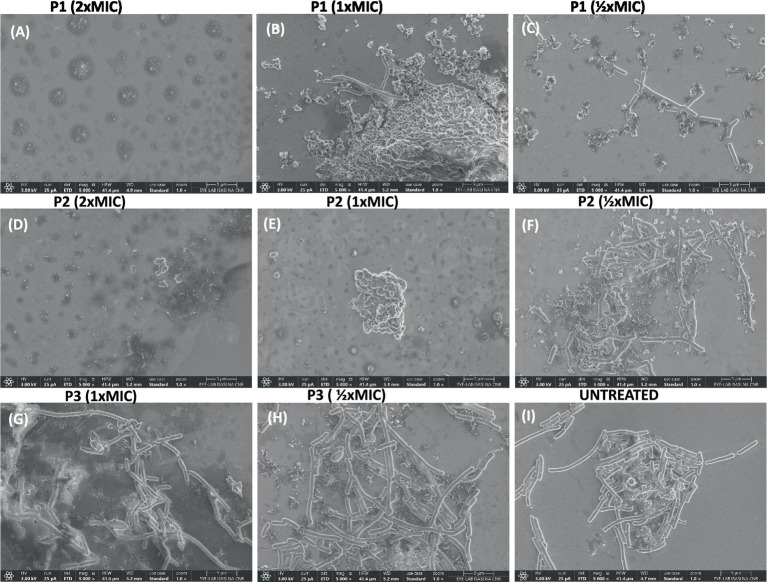
SEM analysis of *K. pneumoniae* cells treated with different peptide concentrations. **(A–C)** Bacteria treated with pantinin-1, **(D–F)** bacteria treated with pantinin-2, **(G,H)** bacteria treated with pantinin-3, **(I)** untreated bacteria.

The effect of peptides on the bacterial morphology was evaluated by scanning electron microscopy (SEM). We observed a different cell morphology for the peptide-treated bacteria compared to the untreated bacteria (Figure 6). In fact, the surface of the untreated cells (Figure 6I) appeared smooth and regular, and the bacteria showed a bacillus shape. Following exposure to peptides, structural changes and the formation of bumps on the cell wall were evident (Figures 6A–H). By increasing the peptide concentration at MIC and 2 × MIC, accumulation of intracellular material and a cluster of debris due to cellular damage was observed outside the cells. In detail, when bacteria were treated with the higher concentration of pantinin 1 and pantinin 2, no intact cells were present (Figures 6A,D); on the contrary, at 1 × MIC concentration, the bacterial load was strongly reduced (Figures 6B,E).

### 3.7 Effect of pantinins on mature biofilm

Biofilm production confers up to 1,000 times more resistance to antimicrobial agents than planktonic cells. The biofilm formation process includes an initial attachment, microcolony formation, maturation, and dispersion (Li and Ni, 2023). Here, we observed that pantinins were particularly effective on disrupting mature biofilm. After seeding the bacterial cells at an optical density of 0.2 OD/mL, the plate was incubated for 48 h to allow the formation of mature biofilm. Pantinins were added for 24 h, and the biofilm biomass was evaluated by the crystal violet method. The results show that all the peptides caused a reduction in the biofilm biomass of 40% (pantinin 1), 30% (pantinin 2), and 20% (pantinin 3) at the concentration of 50 μM.

### 3.8 Meropenem and pantinins combined effect

**Figure 7:**
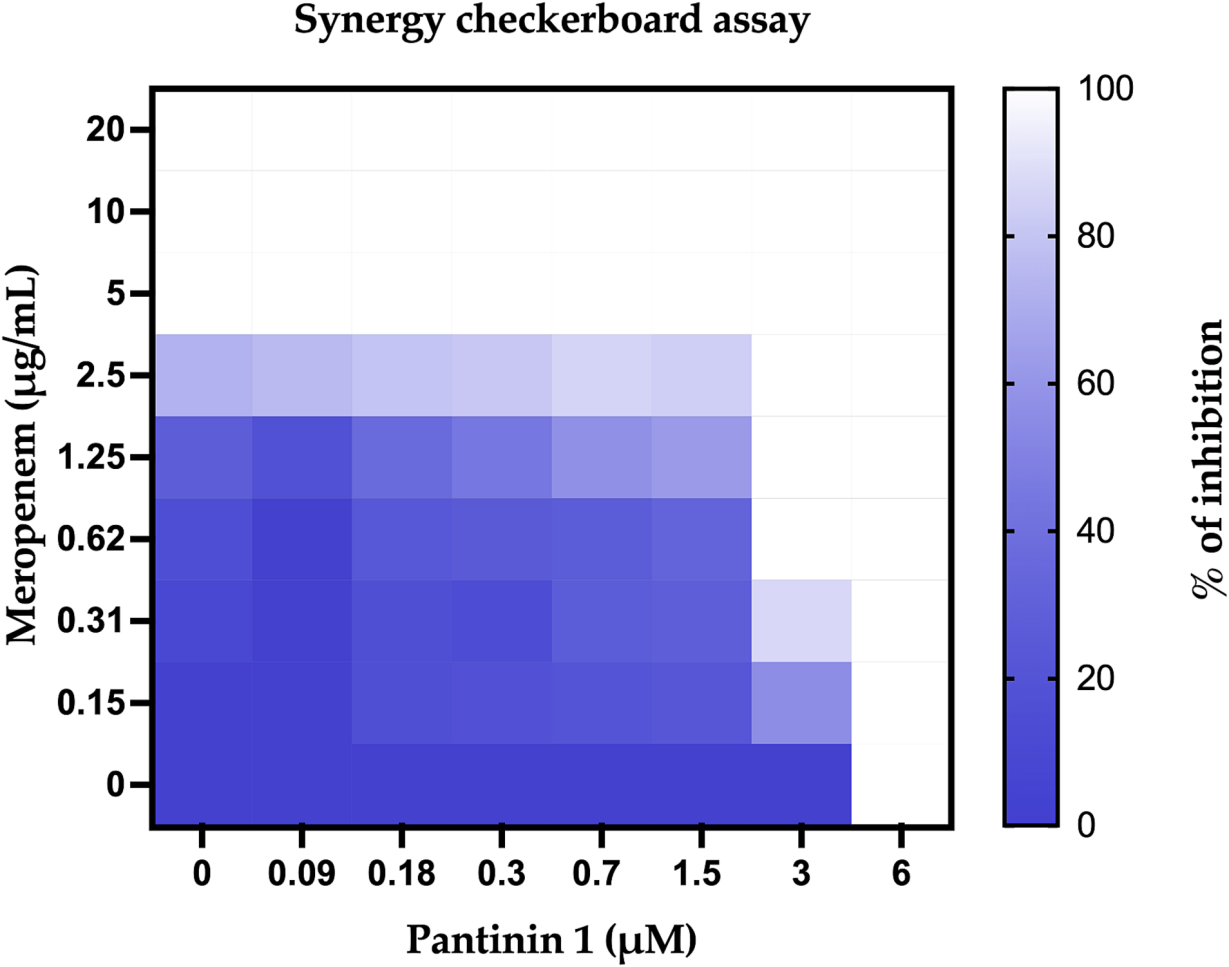
Percentage of bacterial inhibition expressed as heatmap. The columns contain serial 2-fold dilutions of P1, while the rows are presented with 2-fold dilutions of meropenem.

Combination therapy is more effective than monotherapy in fighting severe bacterial infections. The checkboard test investigated the combination of pantinins and meropenem. The results revealed that the MIC value of meropenem against *K. pneumoniae* was 5 μg/mL (Figure 7), while it was reduced to 0.62 μg/mL when combined with 3 μM of pantinin 1. The simultaneous effect of two substances can generate different types of interactions. According to the formula (2), the value of FICI was 0.624, which corresponds to a partial synergy.

### 3.9 Evaluation of virulence factors by real-time PCR

The main factors contributing to the virulence of *K. pneumoniae* include capsule, membrane proteins, fimbriae, siderophores, and ferric iron absorption. We confirmed the ability of pantinins to suppress the expression of representative virulence factors by analyzing some genes such as *galF* involved in the biosynthesis of capsular polysaccharide (CPS), *ompW* encoding for the outer membrane protein W, *fiu* encoding for catecholate siderophore receptor. In particular, pantinin 1 reduced the expression of the virulence genes analysed, showing that it has a role in the inflammatory-modulation mechanism.

## 4 Discussion

One of the main concerns for global public health is the emerging AMR and its burden on the number of deaths and economic losses. *K. pneumoniae,* resistant to beta-lactams and carbapenems, is the leading cause of septicemia, pneumonia, and urinary tract infections (Chatupheeraphat et al., 2023b). These conditions are further worsened by the resistance already developed to the latest antibiotics on the market, such as tigecycline, ceftazidime–avibactam, plazomicin, and eravacycline, (de Souza et al., 2022).

In the present study, we evaluated the antimicrobial action of three peptides derived from the venom of the scorpion *P. pandinus,* namely pantinin 1, pantinin 2, and pantinin 3 (Zeng et al., 2013). These peptides are good antimicrobial candidates since they show a low cytotoxic profile on human keratinocytes (Figure 4) and high antibacterial activity in a very short time up to concentrations of 6.25 μM (Table 4). Accordingly, in previous studies, these peptides showed selectivity toward the breast adenocarcinoma MDA-MB-231 cells and prostate adenocarcinoma DU-145 cells (Zeng et al., 2013), and promising antimicrobial activity against Gram-positive bacteria (Crusca et al., 2018). On the other hand, we demonstrated for the first time that pantinins are also effective against a panel of Gram-negative bacteria, notably against *K. pneumoniae* and its multi-resistant strains, exhibiting higher MIC values (ranging from 25 to 50 μM) compared to the reference strain (ranging from from 6.25 to 25 μM) (Table 5). This broad-spectrum action could be due to the interaction with bacterial membranes via a membranolytic mechanism (de Souza et al., 2022): the cationic nature of peptides could allow electrostatic interactions with anionic lipids in bacterial membranes. Otherwise, this does not occur for eukaryotic membranes that are rich in neutral lipids, explaining the higher concentrations required to cause damage to the cell (Ali and Szabo, 2023; Van Voorst and De Kruijff, 2000). Probably, the decreased antibacterial activity observed for pantinin 3 could be due to a lower propensity to adopt helical conformations in the presence of both TFE (Figure 1) and LPS (Figure 2) compared to pantinin 1 and 2. The bactericidal nature of the three peptides has also been demonstrated via SEM (Figure 6), evidencing the effect of peptides on *K. pneumoniae* surface: at the value of MIC, only a few viable cells with regular morphology are present, while the remaining part shows a jagged and irregular membrane (Figures 6B,E,G). At 2xMIC concentration, cellular and cytoplasmic debris due to cell lysis and increased cellular permeability are observed (Figures 6A,D). Very similarly, Lata et al. (2024) reported that the FKL15 and SKL15 peptides derived from the scorpion *Tityus obscurus* induce damage to bacterial membranes with extracellular extrusions and leakage of intracellular material (Lee et al., 2020).

In general, the antimicrobial potential of peptides secreted by the venom glands of scorpions is well documented; however, few of these AMPs have been evaluated against multidrug-resistant strains (Cesa-Luna et al., 2019; Fong-Coronado et al., 2024; Panayi et al., 2024). Peptides belonging to the same group of pantinins (NDBPs) are potential candidates for the pharmaceutical field due to their high antibacterial but low hemolytic activity. To date, more than 47 short-chain NDBPs derived from various species of scorpions, such as *Mesobuthus martensii*, *Tityus stigmurus*, *P. imperator*, *Androctonus aeneas*, and *Heterometrus petersii,* have been evaluated for their antibacterial properties (Xia et al., 2024). However, only a minority of this group has shown activity against Gram-negative bacteria. Four NDBPs derived from the venom of the scorpion *Urodacus yaschenkoi* showed antibacterial activity against Gram-positive and limited activity against Gram-negative strains. For example, the peptide UyCT2 exhibited MIC values > 32 μM against *P. aeruginosa* and 4 μM against *S. aureus.* Notably, this group of peptides have no activity against *K. pneumoniae*.

**Figure 8:**
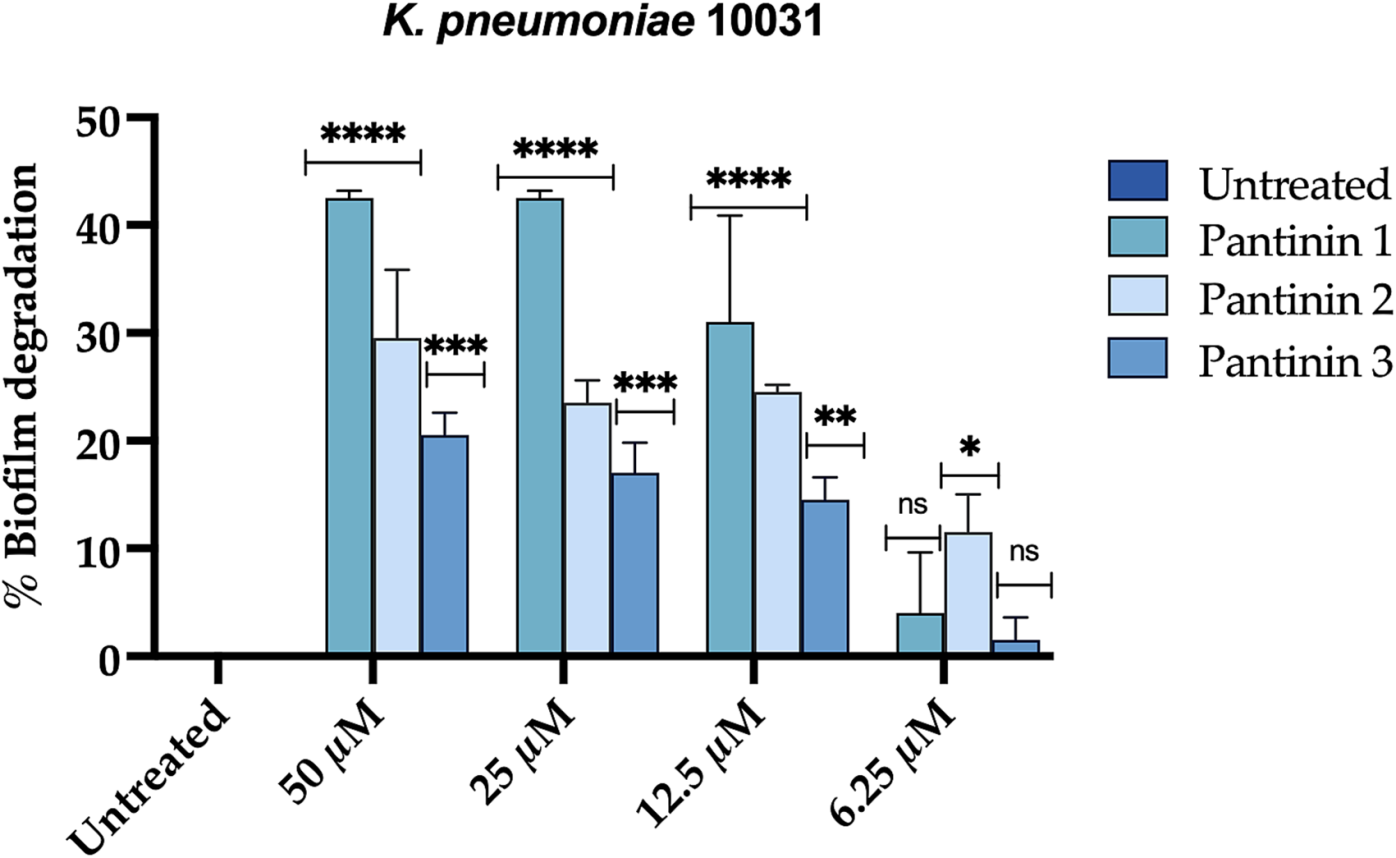
Percentage of degradation of *K. pneumoniae* mature biofilm. Two-way ANOVA with Dunnett’s test for multiple comparisons was performed for statistical analysis. Significances are referred to the untreated sample. ****p < 0.0001, ***p = 0.0002 ** p = 0.021, * p = 0.031, ns (not significant).

**Figure 9:**
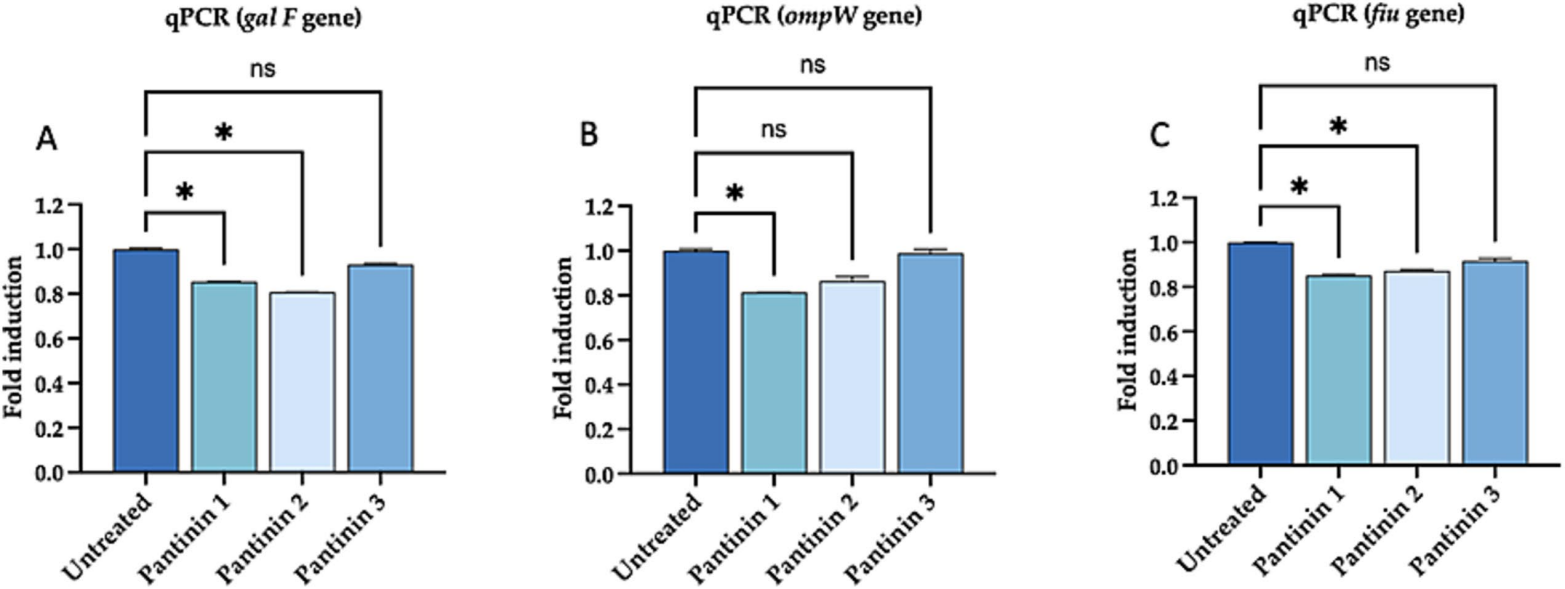
Reduction of gene expression levels determined by real-time PCR. **(A)** *galF* gene coding for the capsular structure **(B)** *OmpW* gene coding for outer membrane protein W, **(C)** *fiu* gene encoding for catecholate siderophore receptor of *k. pneumoniae.* One-way ANOVA was performed for statistical analysis. Significances are referred to the untreated sample, * *p* = 0.031, ns (not significant).

Unlike short-chain NDBPs, long-chain NDBPs (ranging from 41 to 49 amino acids) are more effective against Gram-negative than Gram-positive bacteria. For example, hadrurin, discovered in the mexican scorpion *Hadrurus aztecus*, exhibited potent antibacterial activity at low concentrations against Gram-negative bacteria, including *S. typhi*, *K. pneumoniae*, *P. aeruginosa* and *E. coli* (Torres-Larios et al., 2000). In light of this, our results are very exciting as the pantinins exhibited strong antibacterial activity against *K. pneumoniae* clinical isolates. Moreover, the tendency to form biofilms also provides protection against the immune system and antibiotic resistance, which can be 10–1,000 times greater than in the planktonic form (Surekha et al., 2024). As far as we know, very few studies have evaluated the antibiofilm activities of scorpion peptides. Almaaytah et al., reported the antibiofilm activity against *P. aeruginosa* of Mauriporin isolated from scorpion *Androctonus mauritanicus.* The peptide was able to reduce bacterial charge in a range from 350 μM up to 500 μM (Almaaytah et al., 2014). The peptide BmKn–22 derived from the venom of *Mesobuthus martensii Karsch* inhibited *P. aeruginosa* biofilm formation and destructed the pre-formed one in a concentration range from 800 to 200 μM, recording a % of inhibition from 22 to 50% and a degradation percentage from 24 to 45% (Teerapo et al., 2019). Considering that the biofilms of *K. pneumoniae* promote adaptive resistance to conventional antibiotics, our results are very exciting, especially for pantinin 1, which recorded a degradation percentage of 35% up to 25 μM (Figure 8). A promising strategy for the treatment of drug-resistant bacterial infections is the combined use of antimicrobial agents. It has been reported that the combination of AMPs with other antibiotics enhances antimicrobial efficacy against resistant bacteria compared to monotherapy (Jahangiri et al., 2021; Wu et al., 2009; Chatupheeraphat et al., 2023b; Zharkova et al., 2019; Mood et al., 2021). Recently Chatupheeraphat et al. reported synergistic effects of the AMP K11 in combination with meropenem, chloramphenicol, rifampicin, ceftazidime, ciprofloxacin, and colistin against several clinical isolates of *K. pneumoniae.* The most effective combination was K11 with chloramphenicol, followed by meropenem, rifampicin, ceftazidime, and ciprofloxacin. In contrast, the combination of K11 and colistin exhibited no synergistic effect against any bacterial isolates tested (Chatupheeraphat et al., 2023a). In our study, we demonstrate the combined effect of the pantinin-1 peptide with other agents for the first time (Figure 7). In particular, both the MIC of pantinin-1 and the MIC of meropenem in combination were lower, demonstrating an improvement in efficacy. Meropenem is an antibiotic belonging to beta-lactam and interferes with the synthesis of peptidoglycan. Indeed, the synergy of the combination with pantinin 1 could be due to the enhanced action on the cell wall (Yang et al., 2023). The combination of antibiotics and AMPs, along with chemical modifications and incorporation into delivery systems, represents a strategy to enhance peptide stability and efficacy in the gastric environment - a key challenge for the oral administration of AMPs (Luong et al., 2020). Zhang et al. reported the *in vitro* efficacy of the peptide pexiganan against *H. pylori*. Howerver, *in vivo* gastric proteolytic activity prevented the complete eradication of *H. pylori*, unlike when pexiganan was administered as PNP (Zhang et al., 2015). In conclusion, in addition to the antimicrobial activity of pantinins against clinical strains of *K. pneumoniae*, this study also evaluated their involvement in biofilm formation processes and demonstrated the ability of pantinin 1 to act synergistically with meropenem against *K. pneumoniae*. Taken together, these results suggest that pantinin-1 could serve as a potential AMP for the treatment of *K. pneumoniae* infections (Figure 9).

## Data Availability

The original contributions presented in the study are included in the article/Supplementary material, further inquiries can be directed to the corresponding author.

## Footnotes

^1^ www.graphpad.com
