# Supplementary material for: The inhibitory potential of three scorpion venom peptides against multidrug-resistant Klebsiella pnemoniae
Source: Front Microbiol. 2025 May 30;16:1569719. doi: 10.3389/fmicb.2025.1569719 (PMC12162962; doi:10.3389/fmicb.2025.1569719)
Supplement: Supplementary file 1 [file Table_1.docx]

Supplementary Material

**The inhibitory potential of three scorpion venom peptides against multidrug-resistant *Klebsiella pneumoniae***

Rosa Giugliano^1^, Roberta Della Marca^1^, Annalisa Chianese^1^, Alessandra Monti^2^, Federica Donadio^3^, Emanuela Esposito^3^, Nunzianna Doti^2^, Carla Zannella^1^, Massimiliano Galdiero^1,4^, Anna De Filippis^1,*^

^1^ Department of Experimental Medicine, University of Campania "Luigi Vanvitelli," Naples, Italy;

^2^ Institute of Biostructures and Bioimaging (IBB), National Research Council (CNR), Naples, Italy;

^3^ Institute of Applied Sciences and Intelligent Systems (ISASI), Naples Cryo Electron Microscopy Laboratory - EYE LAB, National Research Council (CNR), Via Pietro Castellino 111, Naples, Italy.

^4^ Complex Operative Unit of Virology and Microbiology, University Hospital of Campania "Luigi Vanvitelli," Naples, Italy.

* Correspondence to Prof. Anna De Filippis, University of Campania "Luigi Vanvitelli," Naples, Italy; [anna.defilippis@unicampania.it](mailto:anna.defilippis@unicampania.it)


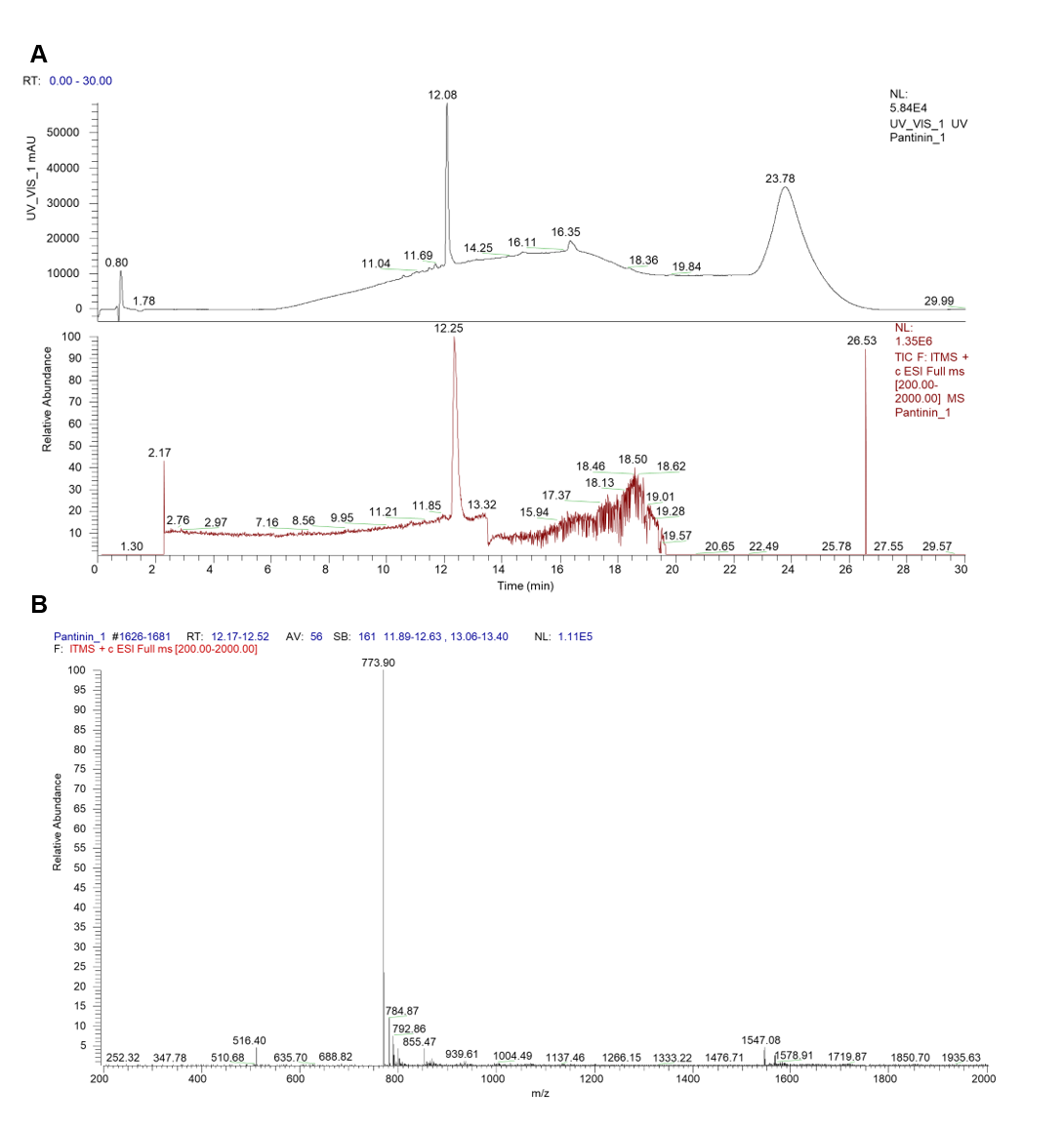


**Fig. S1:** LC-MS characterization of Pantinin 1 peptide. (A) UV-VIS (upper panel) and TIC profiles (lower panel) of Pantinin 1 peptide and (B) MS analysis. The t_R_ value of desired product was 12.08 min and MS analysis showed the expected mass for Pantinin 1 at m/z: 1547.08 ([M+H]^+^) and 773.90 ([M+2H ]^2+^).


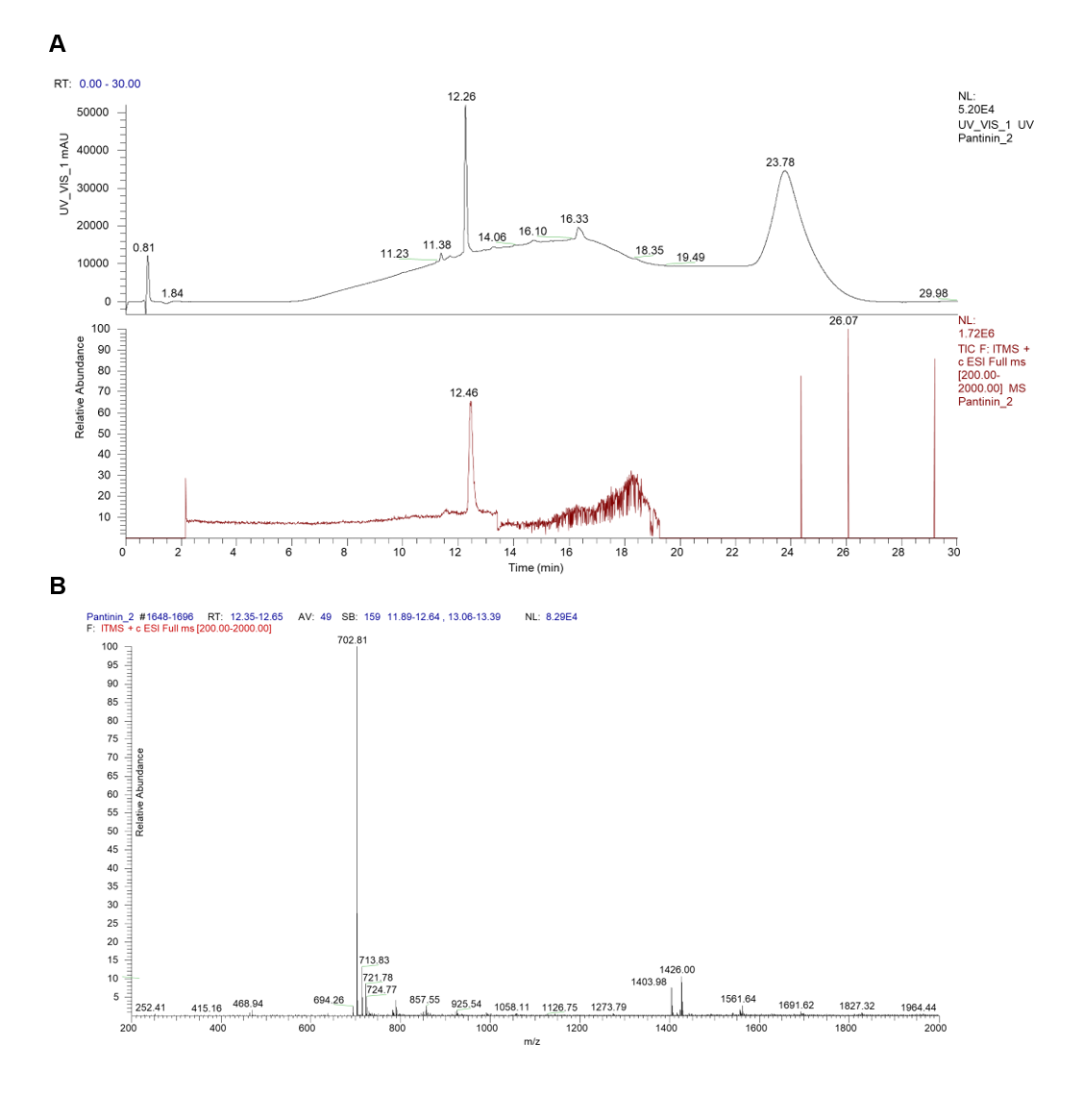


**Fig. S2:** LC-MS characterization of Pantinin 2 peptide. (A) UV-VIS (upper panel) and TIC profiles (lower panel) of Pantinin 2 peptide and (B) MS analysis. The t_R_ value of desired product was 12.26 min and MS analysis showed the expected mass for Pantinin 2 at m/z: 1403.98 ([M+H]^+^), 1426.00 ([M+Na]^+^) and 702.81 ([M+2H ]^2+^).


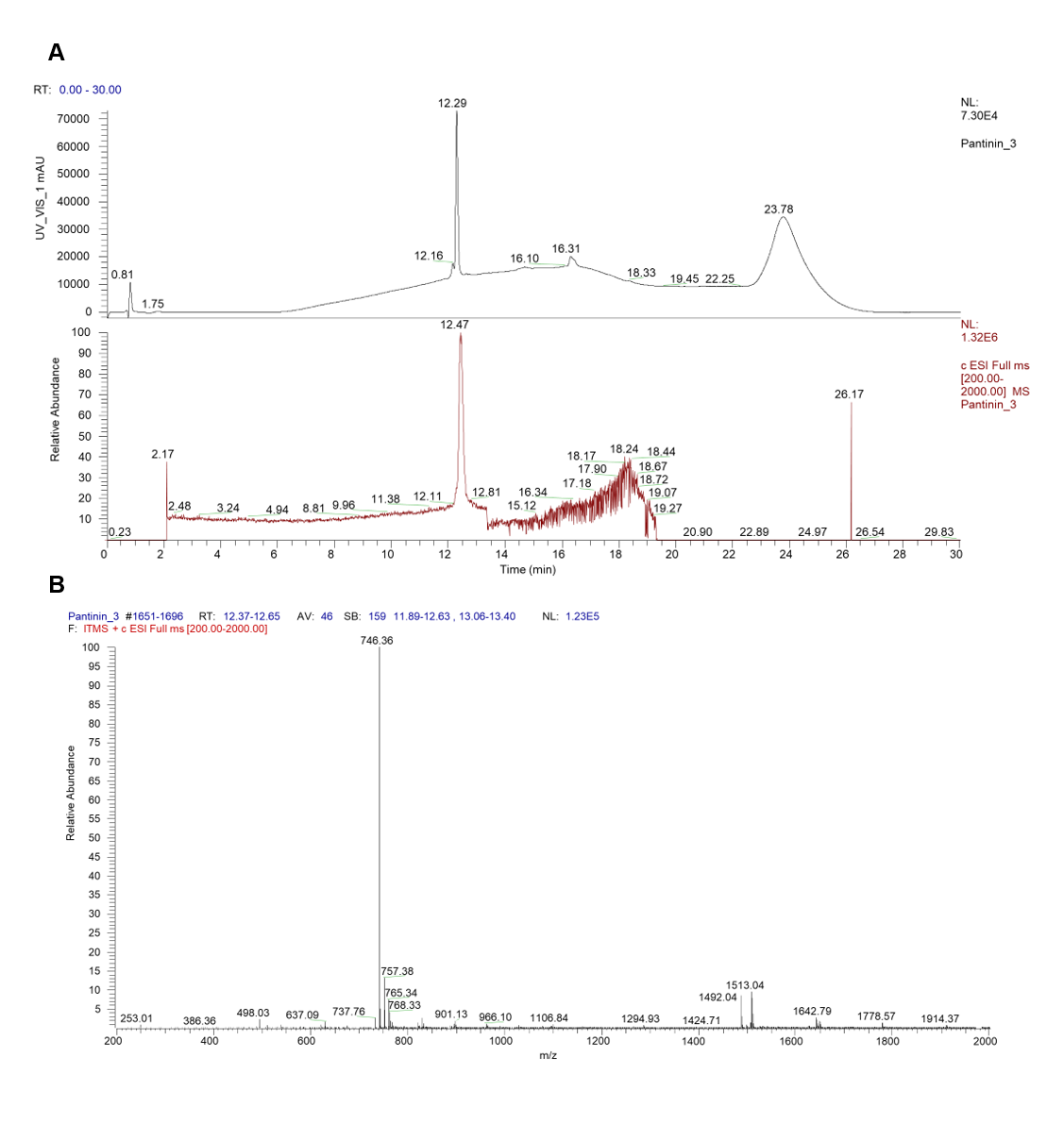


**Fig. S3:** LC-MS characterization of Pantinin 3 peptide. (A) UV-VIS (upper panel) and TIC profiles (lower panel) of Pantinin 3 peptide and (B) MS analysis. The t_R_ value of desired product was 12.29 min and MS analysis showed the expected mass for Pantinin 3 at m/z: 1492.04 ([M+H]^+^), 1513.04 ([M+Na]^+^) and 746.36 ([M+2H ]^2+^).

**
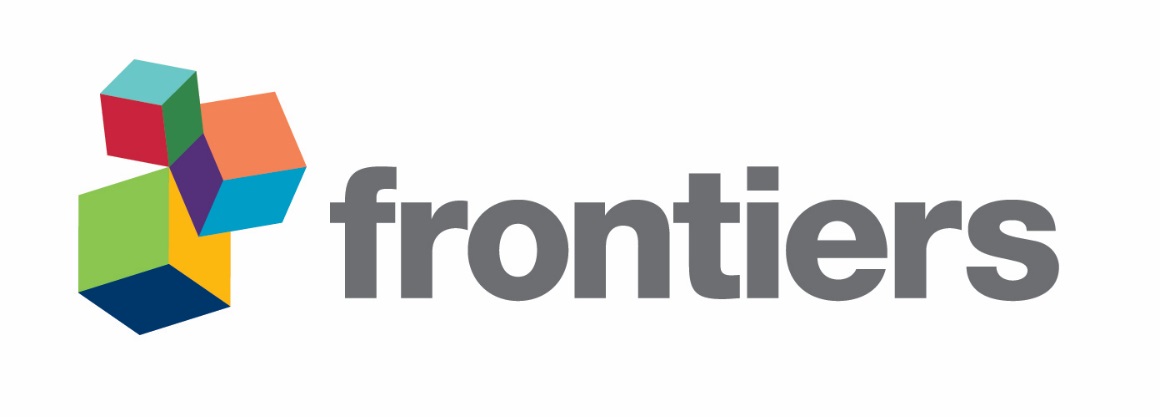
**

.
